# The proteome of the human endolymphatic sac endolymph

**DOI:** 10.1038/s41598-021-89597-3

**Published:** 2021-06-04

**Authors:** Christine Ölander, Jesper Edvardsson Rasmussen, Per Olof Eriksson, Göran Laurell, Helge Rask-Andersen, Jonas Bergquist

**Affiliations:** 1 Department of Surgical Sciences, Section of Otolaryngology and Head Neck Surgery, Uppsala University, Uppsala, Sweden; 2 Department of Chemistry – BMC, Analytical Chemistry, Uppsala University, Uppsala, Sweden

**Keywords:** Anatomy, Biomarkers, Diseases, Medical research, Analytical chemistry, Biochemistry

## Abstract

The endolymphatic sac (ES) is the third part of the inner ear, along with the cochlea and vestibular apparatus. A refined sampling technique was developed to analyse the proteomics of ES endolymph. With a tailored solid phase micro-extraction probe, five ES endolymph samples were collected, and six sac tissue biopsies were obtained in patients undergoing trans-labyrinthine surgery for sporadic vestibular schwannoma. The samples were analysed using nano-liquid chromatography-tandem mass spectrometry (nLC-MS/MS) to identify the total number of proteins. Pathway identification regarding molecular function and protein class was presented. A total of 1656 non-redundant proteins were identified, with 1211 proteins detected in the ES endolymph. A total of 110 proteins were unique to the ES endolymph. The results from the study both validate a strategy for in vivo and in situ human sampling during surgery and may also form a platform for further investigations to better understand the function of this intriguing part of the inner ear.

## Introduction

**Figure 1:**
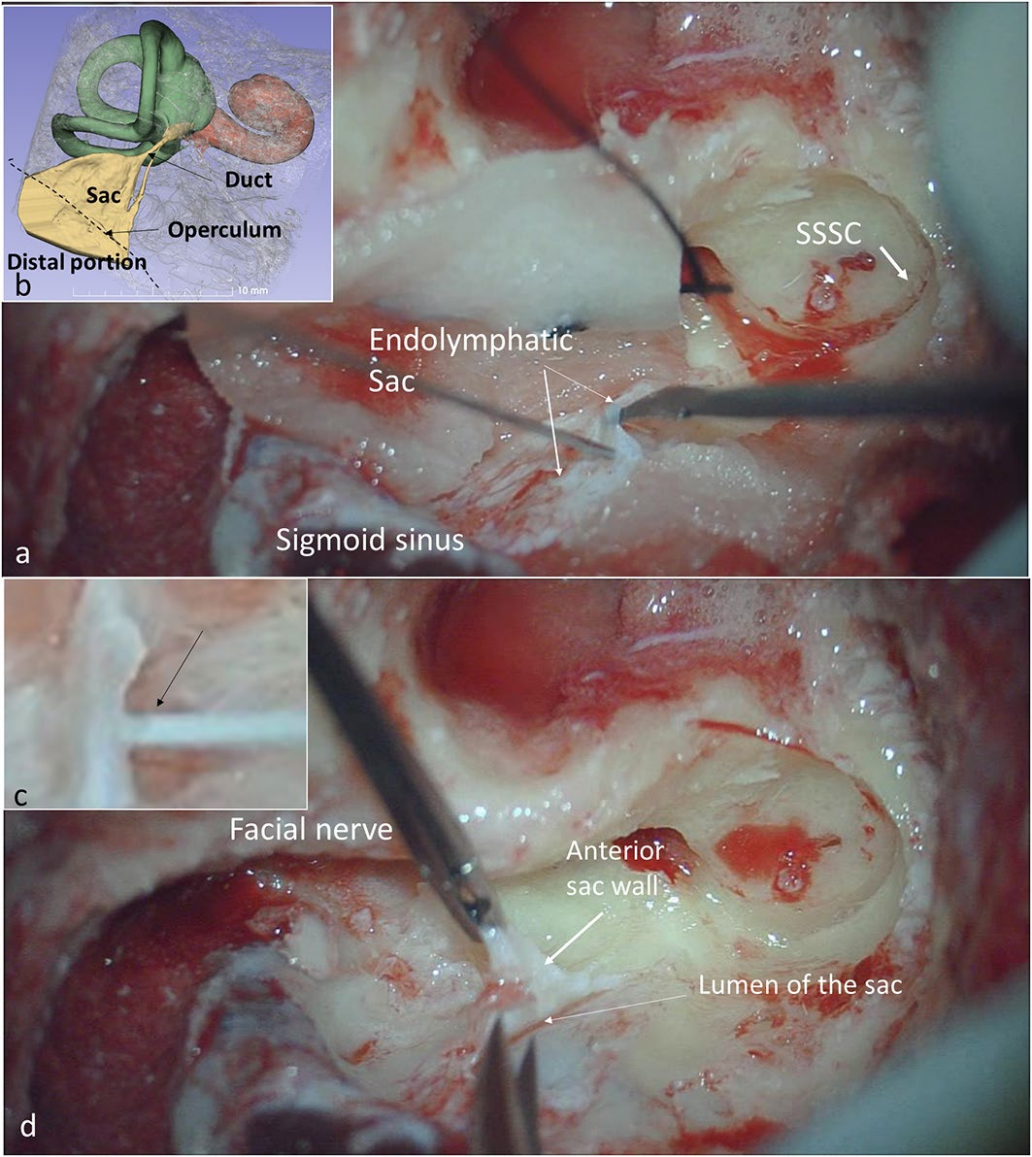
Surgery and biopsy for proteome analysis of the endolymphatic sac. (**a**) During surgical labyrinthectomy the endolymphatic sac is reached from the lateral side. The sac is opened from the duct region in order not to contaminate its content. The sac reaches to the sigmoid sinus. (**b**) Micro-CT of human temporal bone shows the three principal parts of the labyrinth presented in different colours (scale 10 mm). The operculum is the bony ledge separating the intraosseous from the extraosseous part. *SSCC*; superior semicircular canal. (**c**) Collection of ES luminal fluid (arrow) by using the in-situ sampling SPME probe. (**d**) Biopsy of the endolymphatic sac wall is demonstrated.

The inner ear contains two main extracellular fluid compartments, the perilymph and endolymph. They play essential roles in the relay of mechanic-electric transduction of sensory cells important for human hearing and balance. The endolymphatic sac (ES) is the third part of the inner ear, along with the cochlea and vestibular apparatus, and is located in the bony canal of the vestibular aqueduct, reaching a dura mater duplicature in the posterior cranial fossa near the cerebellum. The ES has a direct connection to the rest of the membranous labyrinth through the filiform endolymphatic duct (Fig. 1).

The functions of the ES have been widely discussed over several years. The ES is believed to play a role for the electrolyte homeostasis and endolymph resorption^1^, to regulate endolymph pressure and volume^2,3^, in the elimination of endolymphatic waste products by phagocytosis^4^ and to be involved in immune defence^5^. There have also been suggestions that it may possess endocrine activity^1,6^.

Advancements in mass spectrometry (MS) for the analysis of proteins have greatly improved protein identification. However, thus far, it has not been possible to analyse the human endolymph proteome with MS.

Sampling of the low volume ES endolymph^7^ is technically difficult and entails a high risk of contamination by surrounding tissue, fluids and blood. For pure samples, refined techniques are required. Inspired by the versatility and format of the solid phase microextraction (SPME), as presented by Pawliszyn^8^, the current study was undertaken to explore the usefulness of a miniaturised sampling strategy for clinical in situ sampling of proteins in the ES endolymph. SPME is a solid phase extraction sampling technique that involves the use of a fibre coated with an extracting surface. A solid porous reversed-phase (C18) coating is employed, which attracts hydrophobic regions present in every protein. The format of the probe allows rapid proteome detection with the combination of on-surface enzymatic digestion (oSED)^9^ and high-resolution MS. To the best of our knowledge, this approach has not been previously demonstrated or validated for in vivo and in situ human clinical sampling. Thus, the aim of the study was to demonstrate this novel technique of sampling proteins in small hidden luminal spaces in the human body and to describe the proteome of ES endolymph.

## Results

In total, 1656 different proteins were identified in the 11 samples using MaxQuant software. The full list of proteins identified by MaxQuant is provided in Supplemental Table S1. In the five ES endolymph samples, a total of 1211 different proteins were identified; in the six ES tissue biopsy samples, 1546 different proteins were detected. A total of 1101 of the proteins overlapped and were found in both the endolymph and the biopsy samples. Amongst the 1656 proteins, 110 proteins were exclusively demonstrated for the ES endolymph, and 445 proteins for the ES tissue. Of the 1656 proteins, 197 were detected in all 11 samples. The distribution of proteins in relation to patients and samples can be found in Supplemental Table S2a–c.

By excluding the proteins found in the ES tissue biopsies, we could separate the proteins detected in the ES endolymph, representing the proteins exclusively for the ES endolymph. Eleven of these proteins were detected in all five SPME probe samples (Table 1).

**Table 1:** Proteins exclusively present in all five samples from the ES luminal fluid.

Gene ID	Protein	Gene
P04259	Keratin, type II cytoskeletal 6B	KRT6B
Q5T749	Keratinocyte proline-rich protein	KPRP
P06454	Prothymosin alpha	PTMA
Q5T750	Skin-specific protein 32	XP32
P17661	Desmin	DES
Q13835	Plakophilin-1	PKP1
Q08554	Desmocollin-1	DSC1
P02810	Salivary acidic proline-rich phosphoprotein 1/2	PRH1 & 2
Q6UWP8	Suprabasin	SBSN
Q8N1N4	Keratin, type II cytoskeletal 78	KRT78
Q9NZT1	Calmodulin-like protein 5	CALML5

The proteins were not to be found in any of the samples from the ES tissue biopsy samples*.*

The proteins found in ES endolymph, uploaded to the PANTHER classification system and analysed regarding gene ontology slim (GO-slim) molecular function and protein class, were selected as follows:

1. Proteins found in at least 3 out of 5 ES endolymph samples—a total of 559 proteins, here referred to as ‘ES endolymph general proteins’ (Supplemental Table S3)
2. Proteins found exclusively in the ES endolymph samples and in at least 3 out of the 5 samples—a total of 30 proteins. They are referred to as ‘ES endolymph core proteins’ (Supplemental Table S4).

**Figure 2:**
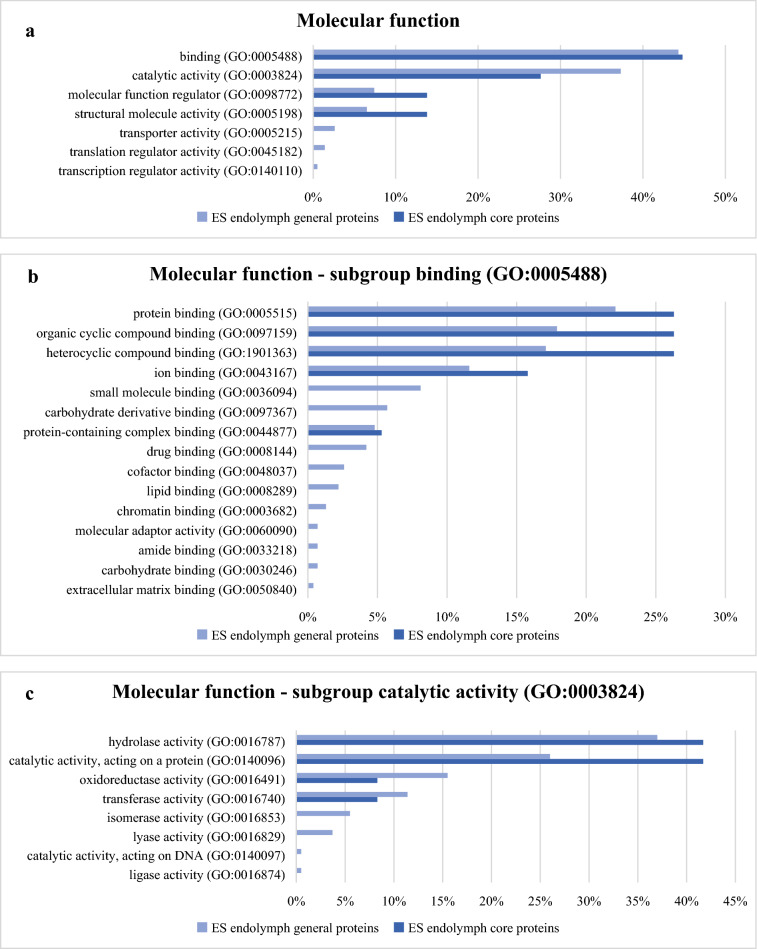
Molecular function according to PANTHER GO-slim amongst ES endolymph general proteins and ES endolymph core proteins. All results presented as a percent of identified proteins out of the total number of protein function hits. (**a**) Main group molecular function; (**b**) in the subgroup binding; (**c**) in the subgroup catalytic activity*.*

**Figure 3:**
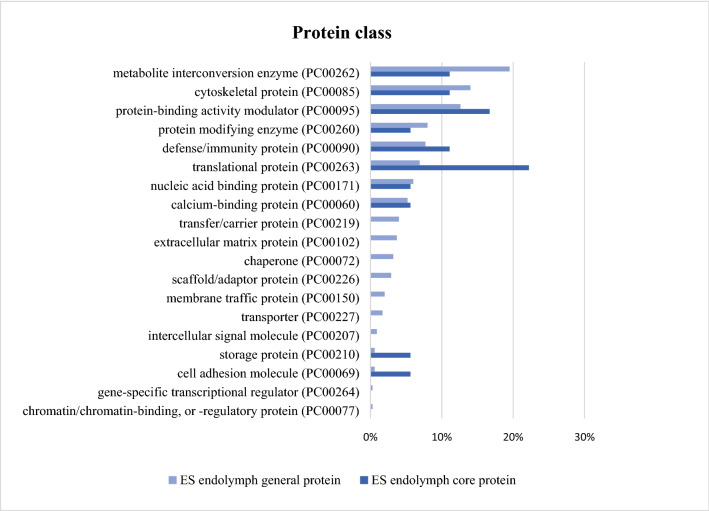
Protein class according to the PANTHER classification system amongst ES endolymph general proteins and ES endolymph core proteins. Results presented as a percent of identified proteins out of the total number of proteins in various classes.

A total of 545 protein IDs were analysed regarding GO-slim molecular function (Fig. 2) and protein classes (Fig. 3). PANTHER was unable to map 14 of the protein IDs since they were not included in the PANTHER protein library.

The 30 ES endolymph core proteins were uploaded to the PANTHER classification system. A total of 28 protein IDs were analysed according to their molecular function (Fig. 2) and protein class (Fig. 3). PANTHER was unable to map two of the protein IDs since they were not included in the PANTHER protein library.

### PANTHER GO-slim molecular function

Amongst the ES endolymph general protein, the PANTHER GO-slim molecular function identified 418 different function hits correlated with the protein IDs (Fig. 2a). Eighty-two percent (82%) of the protein IDs correlated with the molecular function subgroups’ binding and catalytic activity. Fourteen percent (14%) belonged to the smaller subgroup’s structural molecular activity and molecular function regulator.

Out of the 28 protein IDs amongst the ES endolymph core proteins, PANTHER GO-slim molecular function identified 29 different function hits correlated with the protein IDs (Fig. 2a). The most significant portion of molecular functions belonged to subgroup binding (13 protein IDs) and the catalytic activity groups (8 protein IDs), followed by structural molecular activity (4 protein IDs) and molecular function regulator (4 protein IDs).

#### Subgroup binding

In both ES endolymph general proteins and ES endolymph core proteins, subgroup binding (GO:0005488) dominated. There were four main protein groups that excelled: protein binding (GO:0005515), organic cyclic compound binding (GO:0097159), heterocyclic compound binding (GO:1901363) and ion binding (GO:0043167) (Fig. 2b).

In the ES endolymph general protein group, 185 proteins were detected taking part in 457 molecular function hits in subgroup binding.

Protein binding (GO:0005515) represented 22% of the protein IDs in subgroup binding. Seventy percent (70%) of protein IDs found amongst protein binding (GO:0005515) were found to be part of signal receptor binding proteins, enzyme binding proteins and cytoskeletal binding proteins.

The organic cyclic compound binding (GO:0097159) represented 18% of the protein IDs in subgroup binding. The heterocyclic compound binding (GO:1901363) represented 17% of the protein IDs in subgroup binding. Both groups were to 90% represented by nucleic acid binding proteins and nucleoside phosphate binding proteins.

Ion binding (GO:0043167) represented 12% of the protein IDs in subgroup binding. In this group, 42% were cation binding, and 58% were anion binding proteins.

In the more focused ES endolymph core protein group, 13 proteins were detected taking part in 19 different molecular function hits in subgroup binding. Five protein IDs were found in the protein binding, dominated by enzyme binding, which was mostly protease binding. Five protein IDs amongst the organic cyclic compound binding and five protein IDs amongst the heterocyclic compound binding were found. Here, nucleic acid binding represented 100% of the protein IDs in both groups. Three protein IDs amongst ion binding were found, which were represented 100% by cation binding proteins (metal ion binding).

#### Subgroup catalytic activity

The subgroup catalytic activity was the second largest group amongst both the ES endolymph general proteins and ES endolymph core proteins. There were four main protein groups that excelled: hydrolase activity (GO:0016787), catalytic activity, acting on a protein (GO:0140096), oxidoreductase activity (GO:0016491) and transferase activity (GO:0016740) (Fig. 2c).

In the ES endolymph general protein group, 156 proteins were detected taking part in 219 molecular function hits. A total of 37% of the catalytic activity proteins were represented by hydrolase activity (GO:0016787). The hydrolase activity group consisted of 82% of peptidase and hydrolase proteins (acting on acid anhydrase and acting on acid phosphorus–nitrogen bonds).

The catalytic activity acting on a protein (GO:0140096) represented 26% of the protein IDs in subgroup catalytic activity. This group was dominated by peptidase proteins. Oxidoreductase activity (GO:0016491) represented 16% of the protein IDs, and transferase activity (GO:0016740) represented 11% of the protein IDs.

Eight proteins in the ES endolymph core protein group were detected, taking part in 12 molecular function hits. These groups of proteins consisted of five proteins representing hydrolase activity (GO:0016787) and five proteins representing catalytic activity, acting on a protein (GO:0140096). Both groups were dominated by peptidase activity proteins. One protein represented transferase activity (GO:0016740), and one protein represented oxidoreductase activity (GO:0016491).

#### Subgroup molecular function regulator and structural molecular activity

The third and fourth biggest subgroups in both ES groups were represented by molecular function regulator and structural molecular activity (Fig. 2a).

In the ES endolymph general protein group, 31 proteins in total took part in 31 function hits regarding molecular function regulators (GO:0098772). Molecular function regulators consisted of 97% of enzyme regulator activity, mainly proteins with peptidase inhibitory activity.

The structural molecular activity (GO:0005198) was represented by 27 proteins that took part in 19 function hits related to structural constituents of ribosomes.

Amongst the ES endolymph core proteins, four took part in four function hits regarding molecular function regulator (GO:0098772), and four proteins took part in four function hits regarding structural molecular activity (GO:0005198). The molecular function regulator proteins were enzyme regulator activity proteins that were represented mostly by proteins with peptidase inhibitor activity, and the structural molecular activity proteins were represented by the structural constituents of ribosome proteins.

### PANTHER protein class

Amongst the ES endolymph general proteins, 349 protein classes were found according to the PANTHER protein class, which were divided into 19 different categories. The three most prominent categories were metabolite interconversion enzyme (PC00262), cytoskeletal protein (PC00085) and protein binding activity modulator (PC00095) (Fig. 3).

Amongst the ES endolymph core protein 28 protein IDs, 18 protein classes were found according to the PANTHER protein class, which were divided into 10 different categories. The two most prominent categories were translational protein (PC00263) and protein binding activity modulator (PC00095).

## Discussion

The human ES is a thin structure with a low volume hidden in the posterior surface bone of the petrous pyramid in the skull base. Its concealed location complicates the study of ES fluid. In this explorative study, we were able to extract ES endolymph proteins using a refined SPME technique demonstrated for the first time and validated for in vivo and in situ human samplings.

Altogether, 1656 proteins were found, and 1101 of the proteins overlapped between ES tissue and ES endolymph. The most common proteins in ES endolymph samples, found in at least 3 out of 5 samples, were 559 proteins. By excluding proteins that overlapped in the ES tissue and endolymph, 110 proteins exclusive to ES endolymph were detected. That a large number of unique proteins could be identified in the ES endolymph suggests that the sampling technique was successful in the selective attraction of proteins from the ES endolymph without contamination. The ES endolymph core proteins were presented as proteins found in at least 3 out of 5 of these fluid samples, resulting in 30 proteins, a subgroup of 5.4%.

The PANTHER classification systems were employed to classify the molecular functions and protein classes of identified proteins^10^. The same classification system has been used before in similar studies on human perilymph^11,12^. To elucidate the proteome of the ES endolymph general proteins and the ES endolymph core proteins, the purified version of the ES endolymph core proteins may be the best way to represent more specific ES endolymph proteins. Highlighting only the ES endolymph general or core proteins, as was done in this study, does not exclude the possibility that less frequently identified proteins might be equally or even more important in the understanding of the function of the ES. To interpret the data, some methodological aspects must be considered related to the existing knowledge about proteins that are exclusively cytosolic and plasma membrane bound and proteins that may be detected in extracellular fluids. When analysing tissue proteomics, we expect to see a complex mixture of membrane-bound, intracellular and soluble proteins, while when analysing extracellular material, we expect to see an enrichment of soluble and excreted proteins. It is well known that different parts of the ES have different morphology and cellular expression^13^. The samples were taken from the intraosseous region of the ES which is believed to be metabolically active and representative. The size of the ES vary significant between individuals, therefore will the loci for the biopsy sample have a inherit variation and possibly effect the result.

The molecular function of proteins was dominated by binding, catalytic activity, structural molecular activity and molecular function regulator according to GO profiles and classifications.

The same pattern has also been seen in proteomic studies of perilymph, cerebrospinal fluid (CSF) and serum^11^.

A small number of differences between ES endolymph general proteins and ES endolymph core proteins were identified. Amongst the organic cyclic and heterocyclic compound binding proteins, there were only nucleic acid binding proteins in the ES endolymph core proteins. Amongst the ES endolymph general proteins, nucleoside phosphate binding proteins were also well represented. Amongst the proteins found in the ion binding group, the anion binding proteins were the largest group in the ES endolymph general proteins. However, amongst the ES endolymph core proteins, exclusively cation-binding proteins could be detected. Their ability to bind for example Ca2+ and K+ could hypothetically contribute to homeostatic stability in the ES.

PANTHER protein class analyses showed many similarities in the distribution of the proteins. Amongst the ES endolymph core proteins, PANTHER classification system demonstrated a high percentage of protein classes related to binding-function and translational function which could be a part of the supposed stabilising function of the ES. Also, the defence/immunity proteins are represented with about 10% of the proteins detected among the ES endolymph core proteins. This may be consistent with other studies describing the immunological activity in the ES^5^.

A few studies were recently presented on the proteome of human perilymph in cochleae affected by different pathological conditions^11,12,14^. The protein concentration in perilymph is higher in VS patients compared to CI patients^11,14^.

The endo-cochlear potential is the main driving force for sensory transduction and depends on a fine regulation of ion channel activity and transporter proteins in the lateral wall of the cochlea. The regulation of endolymph electrolytes has been well characterised in animal models^1^, but less is known about electrolyte content and secretory activity in human. Solé et al.^15^ demonstrated the proteome of the endolymph in cuttlefish using two-dimensional differential in gel electrophoresis (2D-DIGE) and MS. Although animal models may give important information about endolymph and inner ear function, the results may not be entirely translated to humans. By applying histochemical methods, experiments on animals demonstrated that the ES endolymph is filled with a stainable substance consisting of macromolecular complexes and proteoglycans^16,17^. We identified at least 14 different glycoproteins amongst the 1656 detected proteins. They were well represented in all five ES endolymph samples. Hyaluronan binding proteins, essential for osmotic regulation and water transport, could not be verified in any of the fluid samples. The role of an endocrine capacity of the ES for fluid absorption and/or secretion function has been speculated upon. We did not, however, detect any mineralocorticoid receptor proteins, but one corticosteroid binding globulin was present in one out of five ES endolymph samples. Angiotensinogen was represented in four out of five endolymph samples. Angiotensinogen is known to be well represented in CSF^18^. It can be speculated whether angiotensinogen is involved in pressure regulation and endolymph volume homeostasis.

Among the eleven unique proteins detected in ES luminal fluid (Table 1) both Salivary acidic proline-rich phosphoprotein 1/2 and Calmodulin-like protein 5 may take part in the Ca-regulation. Salivary acidic proline-rich phosphoprotein is a highly potent inhibitor of crystal growth of calcium phosphates^19^. Subgroups of Calmodulin-like protein 5 are Ca-binding proteins which prior to this, in human studies, was only found in epidermis^20^.

Among the eleven proteins exclusively found in the ES luminal fluid desmin was represented. This protein has earlier been shown to be expressed in pericytes in the intrastria vascularis microvasculature^21^. Amongst the ES endolymph core proteins, exclusively cation-binding proteins could be detected. Their ability to bind for example Ca2+ and K+ could hypothetically contribute to maintaining the barrier function of the ES.

Among the 11 proteins exclusively present in all five samples from the ES luminal fluid, three of the proteins are related to keratin. Keratin is often related to epithelial cells and can be a sign of contamination although these proteins were found exclusively enriched in the ES luminal fluid samples. These three isoforms still have an unknown localised function in the ES. Edvardsson Rasmussen et al. presented the proteome of perilymph in VS patients^12^. None of the eleven unique proteins detected in the ES luminal fluid was found in the perilymph proteome which further highlight their unique origin and function. Many proteins, e.g. angiotensinogen, is known to be well represented in several body fluids and tissues, like CSF, but also in perilymph and plasma.

During the microsurgical procedure, when the ES was sampled, the dura mater was intact, and no signs of cerebrospinal fluid was detected. To specifically eliminate the possibility of CSF contamination we also cross checked for brain-specific proteins known to be enriched in CSF^22,23^. None of these proteins were to be found in the ES luminal fluid samples, suggesting a minor risk of contamination.

The electrolyte concentration of the various endolymphatic compartments in the membranous labyrinth is thought to be different in human even though they communicate with each other through the endolymphatic duct and reunion duct. Thalmann et al.^24^ presented the protein profile of the ES endolymph in the guinea pig, and showed that it is more protein rich compared with cochlear and vestibular endolymph. The potassium concentration was significantly higher in cochlear endolymph compared with ES endolymph^25,26^. Moreover, ES endolymph was found to be more acidic (pH 6.7)^27^ than cochlear endolymph (pH 7.4)^25^. Active transport of electrolytes across the ES epithelium may be essential for the regulation of endolymph volume and electrolyte balance. Kämpfe Nordström et al.^28^ demonstrated an immune histochemical rich expression of Na/K-ATPase in the human ES epithelium using super-resolution structured illumination microscopy (SR-SIM). We found Na/K-ATPase α1 to be well represented in four of five ES luminal fluid samples. Subunit β 1 was well represented in six biopsy samples, but only sparsely in the luminal fluid samples. Carbonic anhydrase (CA) is suggested to take part in the acid–base balance in the ES and was found in isoforms 1 and 2 in all five ES endolymph samples.

Our results give further indications that the human ES plays an important role in fluid and ion transport. Several binding proteins that may be essential for trapping cations and transportation are identified in our samples. Their possible role for endolymph absorption and secretion remains to be studied. Different parts of the ES assumingly have different functions. The extra-osseous part contains regions of high cellular activity (intermediate part) while the most distal part of the ES, both in mice and men, is often flat with a rather collapsed lumen. This bellow-like part may act both as an ion transporting region^29^ and as a reservoir potentially playing a role in pressure regulation.

Several studies have suggested that the ES plays an essential role as an immunologic defence organ^30^. The anti-inflammatory factor fetuin-A, known to act as a negative acute phase protein, was present in all five ES endolymph samples. It can be found in both extracellular and intracellular compartments. Fetuin-A has been suggested as a possible independent biomarker for tumour-associated hearing loss in patients with VS^12^. This may indicate that the ES endolymph is de facto affected by the VS.

A recently published review and meta-analysis presented eight proteins exclusively associated with the inner ear that may be candidates as potential biomarkers in the future^31^. Besides cochlin, which was found in 3 out of 5 ES endolymph samples, the eight proteins were detected sparsely in the present study.

Our results are based on a limited number of patients with VS. Tissue and liquid biopsy sampling is an invasive procedure and a potential source of considerable variability. Findings from sampling of perilymph and endolymph have demonstrated a high risk of contamination^32^. Thus, we need to consider that sampling of ES endolymph proteins requires the exposure of the ES in the duplicature of dura mater. It must be acknowledged that the samples could have been contaminated from surrounding tissues, fluids and blood. To reduce this risk, the area for sampling was first thoroughly rinsed with Ringer’s acetate solution before final drilling and opening of the vestibular aqueduct, and the sampling probe was covered with a metal sleeve that was removed as the probe was inserted into the ES.

The results from this explorative study reveal several proteins in the mature ES endolymph involved in protein-binding, homeostatic stabilising, immune reactivity and electrolyte transport. Some of these proteins should be scrutinized further and may give new insight into ES-associated changes such as Meniere’s disease and Large Vestibular Aqueduct Syndrome^29,33^.

Due to the limited number of included patients in this explorative study, we only made comparative analysis between ES tissue and ES endolymph and not between individual samples. The lack of possibilities to obtain control samples is a major limitation of the study that may affect the present findings. Hopefully, further clinical research can be inspired by these results and lead to the inclusion of larger cohorts that would allow for network-based statistical study.

## Conclusions

An in vivo and in situ sampling probe technique was successfully applied and validated for the analysis of proteins in the studied low volume part of the human inner ear. A total of 110 proteins were demonstrated exclusively in the endolymph samples, and 30 of these proteins were found in at least 3 of 5 samples. The results indicate that this tailored sampling techniques provide a novel way to reach new areas of the human body for in vivo analysis. They also present new knowledge on ES endolymph composition and its role in inner ear physiology and eventually lead to a better understanding of inner ear pathophysiology.

## Material and methods

### Patient characteristics

Six patients undergoing trans-labyrinthine surgery (TLS) for sporadic VS were included. Surgeries were performed at the Department of Neurosurgery, Uppsala University Hospital, Sweden. Patients with previous neurosurgery, middle ear surgery, radiation therapy or significant comorbidity were not included in the study. There were three male and three female patients with a median age of 43 years (range 35–65 years) (Table 2). All patients underwent magnetic resonance imaging (MRI) prior to surgery, and the size of the tumour was assessed. Hearing level was defined according to PTA4 (the average of the patient’s air conductive hearing thresholds, dB hearing level ([dB HL]), at four tested frequencies, 500, 100, 2000 and 4000 Hz) in the VS affected ear and the unaffected ear. Samples were collected from the ES wall and ES luminal fluid. One luminal fluid sample failed during sampling due to contamination and had to be excluded from the study. Five ES luminal fluid samples and six ES biopsy samples were included in further analysis. This study was approved by the Regional Ethical Review Board in Uppsala (Dnr 2013/255). Oral and written informed consent was obtained from all patients prior to surgery. The study adhered to the rules of the Declaration of Helsinki.

**Table 2:** Patients’ demographics.

	Tumour size (mm)	Operated side	Sample luminal fluid	Sample sac biopsy	Hearing affected ear (dB HL)	Hearing unaffected ear (dB HL)
1	37	Left	Yes	Yes	35	5
2	44	Right	No	Yes	19	3
3	25	Right	Yes	Yes	30	11
4	19	Left	Yes	Yes	50	38
5	27	Right	Yes	Yes	Missing	Missing
6	27	Right	Yes	Yes	36	8

### Sampling of ES luminal fluid and ES biopsy

TLS includes a radical mastoidectomy and removal of the neighbouring bone covering the middle fossa dura and the posterior fossa dura, where the ES is located. To identify the ES, the imaginary Donaldson’s line, drawn over the lateral semicircular canal and bisecting the posterior semicircular canal, was used. The line meets the sigmoid sinus, and the sac is located anteriorly to the sinus. This surgical exposure allowed samples to be taken from the ES and ES luminal fluid before the labyrinth was opened.

After bone removal, the surgical field was meticulously cleaned with Ringer’s acetate solution, and haemostasis was carefully controlled to minimise blood and bone-dust contamination. A minimal incision was made in the distal portion of the ES, and an injection needle covering a protein binding silica probe in the format of a solid phase micro extraction device (SPME LC Probe; Supelco, Bellefonte, PA, USA) was introduced into the ES lumen manually (Fig. 1). The injection needle cover was withdrawn, and the probe was left for 5 s and thereafter covered and removed, immediately placed in a sterile vial, frozen in liquid nitrogen, and later transferred to − 80 °C. The human ES is surrounded by a dura duplicate that also extends to some degree into the vestibular aqueduct. We biopsied the ES at its intraosseous region, where the dura constitutes a minimal part of the ES wall. The tissue specimen was immediately placed into a sterile vial, and frozen as described above.

### Preparation and on-surface digestion of on-probe enriched proteins

The sampling probes were sonicated in 100 μL of lysis buffer (8 M urea and 50 mM ammonium bicarbonate [NH_4_HCO_3_]) in a sonication bath for 5 min. Next, 10 μL of 45 mM dithiothreitol (DTT) was added to all samples, and the mixtures were incubated at 50 °C for 15 min, then cooled down to room temperature and 10 μL of 100 mM iodoacetamide (IAA) was added, and the mixtures were incubated for an additional 15 min at room temperature in darkness. Afterwards, 50 μL of 50 mM NH_4_HCO_3_ was added to the samples prior to digestion. Thereafter, the proteins were digested by sequencing grade modified trypsin (Promega Corporation, Fitchburg, WI, USA) at a concentration of 0.1 µg/µL in 50 mM NH_4_HCO_3_ pH 8 overnight at 37 °C. On the following morning, the sample was sonicated for 5 min, and the liquid was collected. The digests were dried down under a vacuum using a Speedvac system (Thermo Scientific, Waltham, MA, USA). The samples were redissolved in 20 µL 2.5% acetic acid and desalted on ZipTip C18 columns (Millipore, Burlington, MA, USA). Briefly, the tip was first wetted in 5 × 10 µL 100% acetonitrile (ACN) and equilibrated with 5 × 10 µL 1% acetic acid. The tryptic peptides were adsorbed to the media using 30 repeated cycles of sample loading. The tip was washed using 5 × 10 µL of 1% acetic acid, and finally, the peptides were eluted in 2 × 10 µL 50% ACN, 1% acetic acid. This procedure was repeated twice. The desalted tryptic peptide eluates were dried down under vacuum and then dissolved in 15 µL of 0.1% (v/v) formic acid prior to nanoliquid chromatography–tandem mass spectrometry (nLC-MS/MS) analysis**.**

### Preparation and on-filter tryptic digestion of the endolymphatic sac

The ES samples were sonicated in 100 μL of lysis buffer (6 M urea and 1% β-octyl glucopyranoside) using a sonication probe for 60 s (3 mm probe, pulse 1 s, amplitude 30%). After homogenisation, the samples were incubated for 90 min at 4 °C during mild agitation. The tissue lysates were clarified by centrifugation for 10 min (16,000×*g* at 4 °C). The supernatant containing extracted proteins was collected and further processed.

The total protein concentration of delipidated proteins was determined using the DC Protein Assay Kit (BioRad Laboratories, Hercules, CA, USA), which is based on the modified Lowry method with bovine serum albumin as the standard^34^. The DC assay was carried out according to the manufacturer’s instructions using a 96-well microtiter plate reader model 680 (BioRad Laboratories).

Aliquots corresponding to 30 μg of proteins were used for digestion. An on-filter digestion protocol was used for tryptic digestion of the samples using 3 kDa filters (Millipore Amicon Ultra 0.5 mL; Burlington, MA, USA). Centrifugation was carried out at 14,000×*g* throughout the protocol. A volume of 10 μL of 45 mM aqueous DTT was added to all samples, and the mixtures were incubated at 50 °C for 15 min to reduce the disulphide bridges. The samples were cooled down to room temperature, and 10 μL of 100 mM aqueous IAA were added before incubating the mixtures for an additional 15 min at room temperature in darkness to carbamidomethylate the cysteines. The samples were transferred to spin filters that had been pre-washed with 250 μL of 20% ACN for 15 min and then 500 μL of water for 20 min. Next, the samples were centrifuged for 10 min to remove the added salts, detergents and other interfering substances. An additional volume of 200 μL of 50 mM NH_4_HCO_3_ in 20% ACN was added, and the filters were spun for 10 min followed by 200 μL of 50 mM NH_4_HCO_3_ and centrifugation for another 10 min. Finally, a volume of 100 μL of 50 mM NH_4_HCO_3_ and 16 μL of sequencing grade modified trypsin (Promega Corporation, Fitchburg, WI, USA) (0.1 μg/μL) were added to the samples. Tryptic digestion was performed at 37 °C overnight. The digests were spun through the filter for 20 min to collect the tryptic peptides. An additional volume of 100 μL of 20% ACN and 1% acetic acid were added, and the filters were spun for 10 min and pooled with the first tryptic peptide filtrate. The collected filtrates were vacuum centrifuged to dryness using a Speedvac system ISS110 (Thermo Scientific, Waltham, MA, USA). The tryptic peptide was redissolved in 60 µL of 0.1% (v/v) formic acid and further diluted four times prior to nano-LC–MS/MS analysis.

### Liquid chromatography (LC)/mass spectrometry (MS) analysis: fluid and sac

The peptides were analysed using a Q Exactive Orbitrap mass spectrometer (ThermoFisher Scientific, Bremen, Germany) equipped with a nano electrospray ion source. The peptides were separated by C18 reversed phase liquid chromatography using an EASY-nLC 1000 system (Thermo Fisher Scientific). A precolumn and analytical column setup was used. The precolumn was a 2 cm EASY column (ID 100 µm, 5 µm particles) (Thermo Fisher Scientific), while the analytical column was a 10 cm EASY column (ID 75 µm, 3 µm particles) (Thermo Fisher Scientific). Peptides were eluted with a 90 min linear gradient from 4 to 100% acetonitrile at 250 nL min-1. The mass spectrometer was operated in positive ion mode, acquiring a survey mass spectrum with a resolving power of 70,000 (full width half maximum), m/z 400–1750 using an automatic gain control (AGC) target of 3 × 106. The 10 most intense ions were selected for higher-energy collisional dissociation (HCD) fragmentation (25% normalised collision energy), and MS/MS spectra were generated with an AGC target of 5 × 10^5^ at a resolution of 17,500. The mass spectrometer worked in data-dependent mode.

The acquired output data from LC/MS were processed using MaxQuant (version 1.6.3.4) software with the Andromeda search engine embedded^35^. The reference for Homo sapiens proteome was extracted from UniProt 2019-01-29, including reviewed and unreviewed annotations^36^. The following parameters were used for data processing: protein FDR 0.01, site FDR 0.01, min. peptide length 7, min. peptides 2, and at least one unique peptide. For the survey scan and MS/MS-scan, a maximum of 20 ppm (Fourier Transformation MS) and 0.05 Da (Ion Trap MS) error tolerances were set. For all searches, the enzyme specificity was trypsin; two missed cleavages were allowed. For protein quantification, the modifications used were oxidation (M), acetylation (protein N-term) and deamidation (NQ). Only proteins with at least two peptides and at least one unique peptide were identified and used for further data analysis.

### Pathway analysis

To describe the relationship between protein sequences and their function, Gene Ontology (GO) analysis was performed using the PANTHER classification system (version 15.0; released 2020-02-14; Gene reference Proteome 2019_04 release)^37^. The GO profiles of the proteins identified were classified in the categories molecular function and protein classes^10^. Panther GO-slim molecular function is defined as the biochemical activity of a specific protein specified on different levels and subgroups, and one protein can be seen in more than one molecular function. The PANTHER protein class uses the different classification systems to sort proteins into a more clinical biologically meaningful group at lesser levels for a more clinically related perspective.

## Supplementary Information

Supplementary Legends.

Supplementary Table S1.

Supplementary Table S2.

Supplementary Table S3.

Supplementary Table S4.

## References

[1] Mori N (2017). Ion transport its regulation in the endolymphatic sac: Suggestions for clinical aspects of Meniere's disease. Eur. Arch. Otorhinolaryngol..

[2] Kim SH (2011). Albumin-like protein is the major protein constituent of luminal fluid in the human endolymphatic sac. PLoS ONE.

[3] Salt AN (2001). Regulation of endolymphatic fluid volume. Ann. N. Y. Acad. Sci..

[4] Rask-Andersen H, Stahle J (1980). Immunodefence of the inner ear? Lymphocyte-macrophage interaction in the endolymphatic sac. Acta Otolaryngol..

[5] Kampfe Nordstrom C, Danckwardt-Lilliestrom N, Laurell G, Liu W, Rask-Andersen H (2018). The human endolymphatic sac and inner ear immunity: Macrophage interaction and molecular expression. Front. Immunol..

[6] Moller MN, Kirkeby S, Vikesa J, Nielsen FC, Caye-Thomasen P (2017). The human endolymphatic sac expresses natriuretic peptides. Laryngoscope.

[7] Buckingham RA, Valvassori GE (2001). Inner ear fluid volumes and the resolving power of magnetic resonance imaging: Can it differentiate endolymphatic structures?. Ann. Otol. Rhinol. Laryngol..

[8] Pawliszyn, J. *Handbook of SPME* (Chemical Industry Press, Beijing, 2009).

[9] Dahlin AP (2012). Multiplexed quantification of proteins adsorbed to surface-modified and non-modified microdialysis membranes. Anal. Bioanal. Chem..

[10] Mi H, Muruganujan A, Casagrande JT, Thomas PD (2013). Large-scale gene function analysis with the PANTHER classification system. Nat. Protoc..

[11] Schmitt HA (2017). Proteome analysis of human perilymph using an intraoperative sampling method. J. Proteome Res..

[12] Edvardsson Rasmussen J, Laurell G, Rask-Andersen H, Bergquist J, Eriksson PO (2018). The proteome of perilymph in patients with vestibular schwannoma. A possibility to identify biomarkers for tumor associated hearing loss?. PLoS ONE.

[13] Bagger-Sjöbäck D, Rask-Andersen H (1986). The permeability barrier of the endolymphatic sac. A hypothesis of fluid and electrolyte exchange based on freeze fracturing. Am. J. Otol..

[14] Lysaght AC (2011). Proteome of human perilymph. J. Proteome Res..

[15] Sole M, Monge M, Andre M, Quero C (2019). A proteomic analysis of the statocyst endolymph in common cuttlefish (*Sepia officinalis*): An assessment of acoustic trauma after exposure to sound. Sci. Rep..

[16] Teichmann I, Vigh B, Aros B (1964). Histochemical studies on gomori-positive substances. I. Examination of the gomori-positive substance in the endolymphatic sac of the rat. Acta Biol. Acad. Sci. Hung..

[17] Erwall C, Takumida M, Bagger-Sjoback D, Rask-Andersen H, Wroblewski J (1989). Uptake of radioactive sulphur in the endolymphatic sac. An autoradiographic study. Acta Otolaryngol..

[18] Sernia C (1995). Location and secretion of brain angiotensinogen. Regul. Pept..

[19] Hay DI, Carlson ER, Schluckebier SK, Moreno EC, Schlesinger DH (1987). Inhibition of calcium phosphate precipitation by human salivary acidic proline-rich proteins: Structure-activity relationships. Calcif. Tissue Int..

[20] Sun BK (2015). CALML5 is a ZNF750- and TINCR-induced protein that binds stratifin to regulate epidermal differentiation. Genes Dev..

[21] Liu YH (2018). Electrophysiological properties of strial pericytes and the effect of aspirin on pericyte K+ channels. Mol. Med. Rep..

[22] Schutzer SE (2010). Establishing the proteome of normal human cerebrospinal fluid. PLoS ONE.

[23] Verbeek MM, De Jong D, Kremer HP (2003). Brain-specific proteins in cerebrospinal fluid for the diagnosis of neurodegenerative diseases. Ann. Clin. Biochem..

[24] Thalmann I, Hughes I, Tong BD, Ornitz DM, Thalmann R (2006). Microscale analysis of proteins in inner ear tissues and fluids with emphasis on endolymphatic sac, otoconia, and organ of Corti. Electrophoresis.

[25] Sterkers O, Ferrary E, Amiel C (1988). Production of inner ear fluids. Physiol. Rev..

[26] Couloigner V, Teixeira M, Sterkers O, Ferrary E (1999). In vivo study of the electrochemical composition of luminal fluid in the guinea pig endolymphatic sac. Acta Otolaryngol..

[27] Tsujikawa S, Yamashita T, Amano H, Kumazawa T, Vosteen KH (1992). Acidity in the endolymphatic sac fluid of guinea pigs. J. Otorhinolaryngol. Relat. Spec..

[28] Nordstrom CK, Danckwardt-Lilliestrom N, Liu W, Rask-Andersen H (2020). "Reversed polarization" of Na/K-ATPase-a sign of inverted transport in the human endolymphatic sac: A super-resolution structured illumination microscopy (SR-SIM) study. Cell Tissue Res..

[29] Eckhard AH (2019). Inner ear pathologies impair sodium-regulated ion transport in Meniere's disease. Acta Neuropathol..

[30] Tomiyama S, Harris JP (1986). The endolymphatic sac: Its importance in inner ear immune responses. Laryngoscope.

[31] Mulry E, Parham K (2020). Inner ear proteins as potential biomarkers. Otol. Neurotol..

[32] Hara A, Salt AN, Thalmann R (1989). Perilymph composition in scala tympani of the cochlea: Influence of cerebrospinal fluid. Hear Res..

[33] Berrettini S (2005). Large vestibular aqueduct syndrome: Audiological, radiological, clinical, and genetic features. Am. J. Otolaryngol..

[34] Lowry OH, Rosebrough NJ, Farr AL, Randall RJ (1951). Protein measurement with the Folin phenol reagent. J. Biol. Chem..

[35] Cox J (2009). A practical guide to the MaxQuant computational platform for SILAC-based quantitative proteomics. Nat. Protoc..

[36] Apweiler R (2017). UniProt: The universal protein knowledgebase. Nucleic Acids Res..

[37] Mi H (2019). Protocol update for large-scale genome and gene function analysis with the PANTHER classification system (v.14.0). Nat. Protoc..

