# Supplementary material for: The proteome of the human endolymphatic sac endolymph
Source: Sci Rep. 2021 Jun 4;11:11850. doi: 10.1038/s41598-021-89597-3 (PMC8178308; doi:10.1038/s41598-021-89597-3)
Supplement: Supplementary file 1 — Supplementary Legends. [file 41598_2021_89597_MOESM1_ESM.pdf]

Supplemental Material

The Proteome of the Human Endolymphatic Sac Endolymph

Christine Ölander[1], Jesper Edvardsson Rasmussen[1], Per Olof Eriksson[1], Göran Laurell[1], Helge Rask-Andersen[1], Jonas Bergquist[2]*

[1] Department of Surgical Sciences, Section of Otolaryngology and Head Neck Surgery, Uppsala University, Uppsala, Sweden

[2] Department of Chemistry – BMC, Analytical Chemistry, Uppsala University, Uppsala, Sweden

*Corresponding author: Professor Jonas Bergquist, email: jonas.bergquist@kemi.uu.se

1) Supplemental Table 1: All 1,656 protein IDs identified by Max Quant and by PANTHER GO-slim.
2) Supplemental Table 2a–c: Distribution of proteins in relation to patients and samples.
3) Supplemental Table 3: 559 proteins representing the ES endolymph general proteins.
4) Supplemental Table 4: 30 proteins representing the ES endolymph core proteins.
