# Supplementary material for: The proteome of the human endolymphatic sac endolymph
Source: Sci Rep. 2021 Jun 4;11:11850. doi: 10.1038/s41598-021-89597-3 (PMC8178308; doi:10.1038/s41598-021-89597-3)
Supplement: Supplementary file 3 — Supplementary Table S2. [file 41598_2021_89597_MOESM3_ESM.pdf]

Supplemental Table 2a-c: Distribution of proteins in relation to patients and samples.

	Total proteins	Unique ES luminal fluid	Unique ES tissue biopsy	Overlapping
**Patient 1**	1,444	48	977	419
**Patient 2**	1,146	--	1,146	--
**Patient 3**	1,114	85	635	394
**Patient 4**	1,117	208	297	612
**Patient 5**	1,131	106	692	333
**Patient 6**	1,309	307	288	714
**Patients 1–6**	1,656	110	445	1,101

a) Protein distribution related to each patient presented as the total amount of proteins in each patient and divided in the number of proteins present only in the ES luminal fluid (unique ES luminal fluid) and only in the ES tissue biopsy (unique ES tissue biopsy), as well as the number of proteins overlapping, i.e. found in both the ES luminal fluid and the ES tissue biopsy samples.

ES luminal fluid samples	Total ES luminal fluid proteins	Unique ES luminal fluid proteins	ES tissue biopsy samples	Total ES tissue biopsy proteins	Unique ES tissue biopsy proteins
**5 out of 5 samples**	234	11	**6 out of 6 samples**	658	60
**4 out of 5 samples**	157	6	**5 out of 6 samples**	194	60
**3 out of 5 samples**	168	13	**4 out of 6 samples**	131	56
**2 out of 5 samples**	272	32	**3 out of 6 samples**	163	86
**1 out of 5 samples**	380	48	**2 out of 6 samples**	176	81
			**1 out of 6 samples**	224	102

b) Distribution of number of proteins according to their presence in the different categories (e.g. ES tissue biopsy sample ‘4 out of 6 samples’ is read as the proteins found in exactly 4 out of the 6 samples).

ES tissue biopsy samples

ES luminal fluid samples

	6/6	5/6	4/6	3/6	2/6	1/6	0/6
5/5	197	8	2	8	1	7	11
4/5	107	13	10	8	4	9	6
3/5	91	20	10	7	14	13	13
2/5	118	31	21	22	22	26	32
1/5	85	62	32	32	54	67	48
0/5	60	60	56	86	81	102	0

c) Distribution of 1,656 proteins according to their presentation in the number of samples found in the ES luminal fluid and in the ES tissue biopsy (e.g. 197 proteins are present in five out of five ES luminal fluid samples [5/5], and the same proteins are also present in all six ES tissue biopsy samples [6/6]).
